# Examining the use of experience sampling methods in sport psychology research: a scoping review

**DOI:** 10.3389/fpsyg.2026.1860704

**Published:** 2026-08-10

**Authors:** Mara L. L. Majer, Mitchell C. Profeit, Luc J. Martin, Jean Côté, Andre Spendlove, Stuart G. Wilson

**Affiliations:** School of Kinesiology and Health Studies, Queen’s University, Kingston, ON, Canada

**Keywords:** ambulatory assessment, daily diary, ecological momentary assessments, experience sampling methods, sport psychology

## Abstract

**Introduction:**

Experience sampling methods (ESMs) allow researchers to capture the variability in momentary human experiences by collecting repeated self-report measures within or across days. Real-time fluctuations in psychological processes, which are commonly distorted by retrospective bias in traditional survey designs, can be more accurately recorded using this approach. ESMs have been widely adopted in health and exercise psychology research, with more recent, sporadic use in the sport context. Given the growing interest in ESMs and their potential impact on the field, this scoping review aimed to characterize the use of ESMs in sport psychology research.

**Methods:**

Following PRISMA-ScR guidelines, we searched four databases for studies conducted in the context of sport, measured a psychological construct, and used ESMs (including daily diaries, ecological momentary assessments, and ambulatory assessments).

**Results:**

The search yielded 1,474 studies, of which 73 were included. The included studies primarily focused on the experiences of sport participants, with limited representation of coaches, parents, or spectators. Most employed daily or twice-daily sampling using fixed or event-contingent schedules, whereas fewer used “traditional” ESM designs (multiple signals per day delivered using semi-random schedules). Studies predominantly examined constructs related to stress and emotion, particularly appraisal and coping pro- cesses. Other thematic research areas included recovery from training, group dynamics, motivation, spectator experiences, comparisons of activity experiences, and medical symptoms.

**Discussion:**

Given the limited use of ESMs and narrow focus on specific contexts, designs, and topics, continued investigation is warranted to better understand critical momentary experiences in sport.

## Introduction

There is considerable variability in day-to-day human experience, which poses challenges for measuring and understanding trends in behaviour, emotion, and other psychological factors. Experience sampling methods (ESMs) aim to measure a participant’s repeated behaviours within their natural setting and record real-time information about their subjective experiences (Shiffman et al., 2008). ESMs collect information about targeted experiences to improve the accuracy and specificity of participants’ reports of the contexts in which those experiences occurred. Because of their capacity to more faithfully represent momentary experiences, ESMs have been used extensively in various domains of psychological and health research (Trull and Ebner-Priemer, 2009). Despite the broad spectrum of dynamic psychological experiences encountered in sport, the application of ESMs in sport psychology remains limited and evolving (Reifsteck et al., 2021), with a lack of clarity regarding the evidence, interventions used, and populations studied.

### Experience sampling and associated methods

ESMs involve repeated questionnaires delivered throughout multiple days or weeks to measure current thoughts, feelings, and emotions about real-world events, environments, and/or relationships (Trull and Ebner-Priemer, 2009; Perski et al., 2022). These methods have become a popular method for data collection in psychological research, as they increase the reliability and validity of measurements by limiting retrospective recall bias (Myin-Germeys and Kuppens, 2022). Specifically, typical cross-sectional or retrospective survey designs ask participants to recall and/or summarize emotions or experiences for a given timeframe (e.g., days, weeks, months). Retrospective recall may be subject to cognitive biases, which overestimate the intensity or importance of an experience (Levine and Safer, 2002). Further, recall that summarizes larger time periods will necessarily aggregate experiences, without representing their temporal or contextual specificity (Myin-Germeys and Kuppens, 2022).

We adopted a broad, inclusive definition of ESMs that encompasses several related terms and methodologies. For example, while *ecological momentary assessments* originated separately from ESMs (Shiffman et al., 2008), these methods pragmatically represent a similar process (Myin-Germeys and Kuppens, 2022). Similarly, whereas traditional ESMs focus on “active” monitoring (e.g., self-report items), *ambulatory assessments* include forms of “passive” monitoring (e.g., sensors, wearable information) that may or may not be paired with active measures (SAA (Society for Ambulatory Assessment), 2024). Finally, although ESMs often involve multiple data collection time points per day, *structured daily diaries* may be considered a form of ESM when used to capture momentary or recent experiences repeatedly. As a result, we conceptualized ESMs as encompassing overlapping methodologies, thereby representing a comprehensive understanding of intensive longitudinal methods.

### Use of experience sampling methods

ESMs have been used extensively in several areas of psychological and clinical/health research (Trull and Ebner-Priemer, 2009). For example, recent reviews have explored the use of ESMs to identify emotional regulation strategies (Boemo et al., 2022), assess emotional and motivational correlates of health behaviours (Votaw and Witkiewitz, 2021; Perski et al., 2022), determine the validity and reliability of ESMs for measuring social dynamics (Langener et al., 2023), and examine how ESMs were used to capture daily levels of well-being, pain, and fatigue in children with chronic disease (van Dalen et al., 2024). Despite their popularity across various psychological and health-related domains, few studies have implemented ESMs in sport psychology (Dickens et al., 2018).

Certain potential barriers may explain the limited use of ESMs in sport. Many sport activities are fast-paced with few opportunities to answer self-report questions in the middle of a play, sequence, or game, which decreases the feasibility of conducting surveys consistently and on time (Reifsteck et al., 2021). The intensity and time–pressure within sport may also exacerbate existing limitations of ESMs. Some athletes may perceive ESMs as intrusive to their preparation for participation and choose to skip responses at certain moments, which may lead to self-selection bias in the types of people represented from collected responses (Cerin et al., 2001). However, these potential barriers are limited to assessing athletes during competitions and practices, which describe only one context within the scope of sport psychology. ESMs may be beneficial for studying the thoughts, emotions, and behaviours involved in processes outside the “arena” (e.g., recovery, group dynamics), or those of other participants involved in the sport experience (e.g., coaches, parents, spectators)—though it is unclear to what extent these avenues feature in current research.

### The current study

ESMs have the potential to more closely represent the momentary experiences of athletes, coaches, parents, spectators, and other actors in and around sport. Despite their potential utility, they have seen limited use in sport psychology (Reifsteck et al., 2021). Therefore, this study used a scoping review design to describe how ESMs have been implemented to study the psychology of sport. Specifically, our objectives were to collect the existing studies that have adopted ESMs in sport to describe (1) the contexts in which ESMs have been implemented (e.g., participants), (2) the study designs that have been used, and (3) the topics that ESMs have been used to measure. By describing how ESMs have been used in sport psychology, this study aimed to identify examples and gaps in the existing evidence to inform future research questions and opportunities.

## Methods

This study used a scoping review design that followed the Preferred Reporting Items for Systematic Reviews and Meta-Analyses extension for Scoping Reviews (PRISMA-ScR) based on previously published protocols (Tricco et al., 2018). We then followed the remaining steps outlined by Sabiston et al. (2022) detailed below: (1) identification of the research question, (2) identification of relevant studies, (3) selection and screening of studies, (4) charting the data, and (5) collating and summarizing information. The full protocol can be found in the Supplementary material or on OSF^1^.

### Identification of the research question

This review followed the broad research question, “How have ESMs been used to study the psychology of sport?” Based on this research question, included articles must have featured original research (i.e., no reviews, commentaries, editorials, etc.) and been published in a peer-reviewed journal (i.e., no chapters or theses). The research must also have involved a sport context, central finding, factor, or characteristic, defined by some form of interaction (e.g., participation, active observation) with sport games, practices, or tournaments. Included articles could compare sport with exercise or physical activity, but could not aggregate them. The research must have investigated a psychological variable, defined as the emergent property of a distributed network of neurons, including cognitions (e.g., beliefs, attitudes, goals), emotions (e.g., negative affect, cravings) and processes operating on these (e.g., self-regulation, learning; Perski et al., 2022). Finally, the research must have involved a variation of ESMs, defined as repeated (i.e., two or more) within-day, daily or weekly self-reported assessments of phenomena within that time frame (Perski et al., 2022). We did not include open-ended diaries, think-aloud protocols, or observation-only methods (i.e., featured no self-perceptions). We also omitted studies that used descriptive experience sampling methods, which involved subsequent interview analysis based on notes recorded in the moment (Dickens et al., 2018; Van Raalte et al., 2019).

### Identification of relevant studies in covidence

An initial search for articles referencing ESMs and sport psychology indicated that several different methods fell under the term ESM, and many articles did not include “ESM” in their titles. Therefore, to incorporate as many relevant studies as possible, we used the following search string built around components of “Sport” and “ESMs”: “sport*” AND (“experience sampl*” OR “daily diar*” OR “ecological momentary assessment*” OR “ambulatory assessment*” OR “ecological momentary intervention*” OR “just in time adaptive intervention*” OR “JITAI*”). After consultation with the institutional Librarian, these search terms were used across four databases: *SportDiscus*, *APA PsychInfo* (via OVID), *Sport Medicine and Education Index*, and *Web of Science*. Databases were first searched on December 27th, 2023; the search was repeated on July 10th, 2024, and December 1st, 2025, and the search results were uploaded to a review management software, Covidence (2026), for article screening.

### Selection and screening of studies

**Figure 1:**
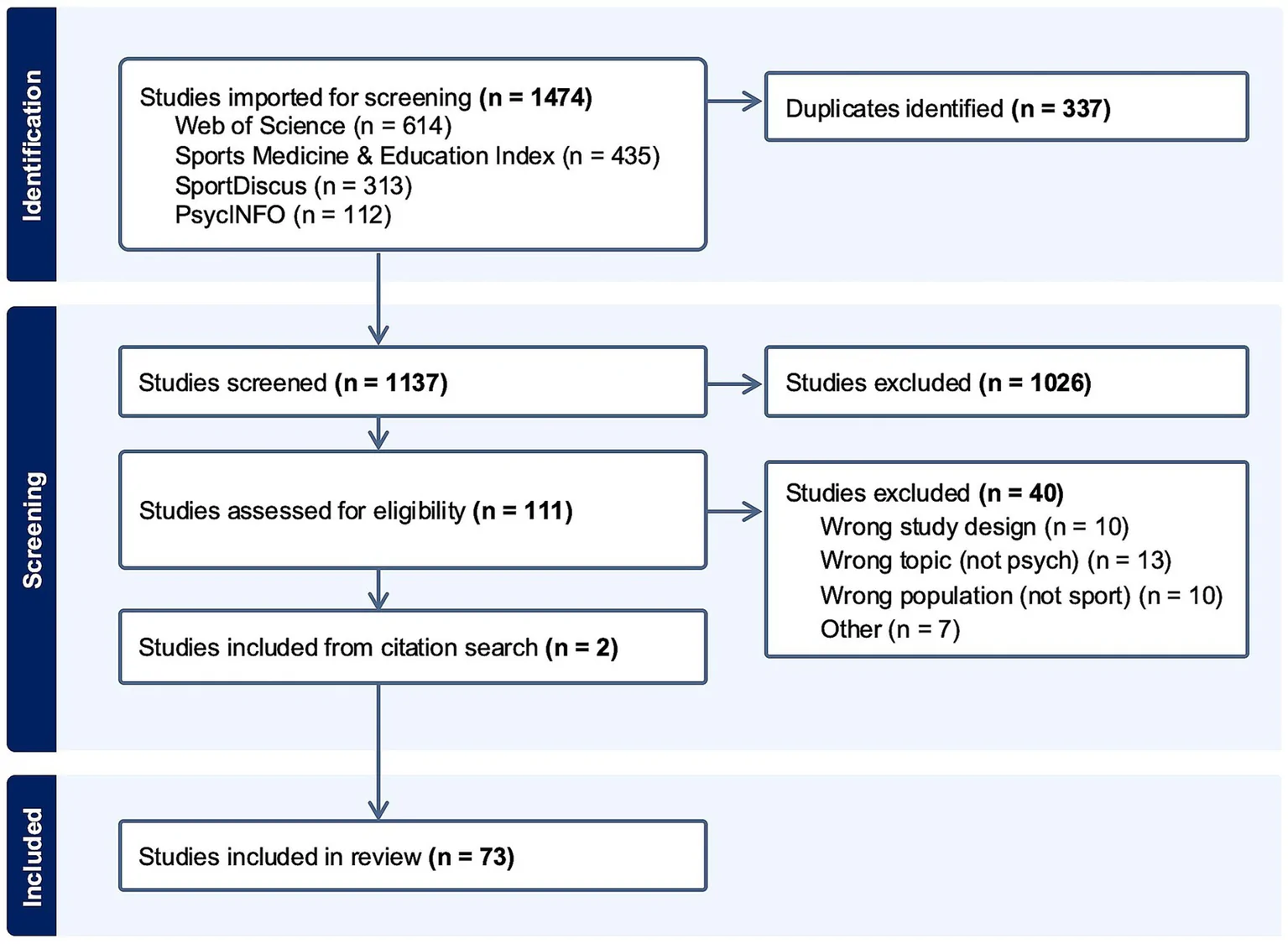
PRISMA-ScR flow diagram of study screening.

The study selection began with a broad search using the included terms in the specified databases. For the final comprehensive search, 1,474 studies were imported for screening (Figure 1). After removing 337 duplicates, 1,137 studies were included in the initial screening of titles and abstracts. Two researchers then independently reviewed the title and abstract of the papers and decided whether to include or exclude them from the review based on the established criteria. One thousand and twenty-six were deemed to have not met the required study topic (i.e., psychology), design (i.e., ESMs), or context (i.e., sport), so 111 were ultimately included in a full-text review. The same two researchers then reviewed the full texts to select which articles followed the inclusion criteria. Forty studies were then excluded from the full-text review based on topic, design, or context. A third researcher from the team addressed any disagreements or discrepancies. Following an expert consultation and review of the reference lists from the initial pool of studies, two additional studies were included in the review, with a final sample of 73 studies (Figure 1).

### Charting the data and collating and summarizing information

A coding sheet was created to collect data points from each study and provide a descriptive summary of the results (the full list of studies and main coding may be found in the Supplementary material or on OSF). The coding terms for the demographic information included the authors, year of publication, title, sample size, location of recruited participants, gender, age, and role of participants (i.e., athlete, parent, coach, spectator), and classifications of sport type and competition level. Further data were collected, outlining the study duration, sampling scheme type, specific questionnaire type, method of data collection, and general area of research. The sampling scheme type was coded as pre-, post-, and/or during competition or a sporting event, along with the frequency of signals throughout the study. Specifically, the frequency of sampling scheme types was classified into four categories: fixed, random, semi-random, and event-contingent. Fixed signals were sent on a regular, predetermined schedule, often in the morning or evening (e.g., each night at 8:00 p.m.). The random scheme had signals sent at any time of day, while a semi-random scheme had signals sent randomly within defined blocks of time (e.g., randomly within five two-hour blocks during the day). The event-contingent schedule had participants complete surveys immediately before/after an event, training session, or competition.

The ESM questionnaire measures were coded as either validated or original, and whether they included open-ended or closed (e.g., Likert-scale) responses. We then determined which studies had previously validated items and scales or subscales, items rationalized by literature, study-specific validity or reliability assessments, no psychometric support, or a combination of these characteristics. The data collection method was coded as online questionnaire, physical, app, or verbal. The included studies were inductively grouped into distinct areas of research according to their psychological topics and research traditions; although some studies could have belonged to multiple areas, these groupings were intended to convey both the breadth and relative interest in various research foci.

## Results

The final analysis included 73 studies, which were coded for the context they described, the methodological features of their design, and the area of research they covered. A complete list of studies and extracted data may be found in the Supplementary material or on OSF.

### Context

Most studies involved athletes—defined as individuals who perform the sport—as participants (*n* = 57). Six of these studies, conducted in school sports or after-school sport programming, labelled the “athletes” as students or sport participants. Two studies had university students complete ESMs as sport participants and sport spectators or viewers (Kim and James, 2019, 2024). The remainder of the studies sampled coaches (*n* = 3), spectators (*n* = 9), coaches and athletes (Balk et al., 2019), or cases of athletes, parents, and a coach (Hayward et al., 2017). Most studies included men and women (*n* = 48), while 17 included only men, 6 only women, and 1 included men, women, and transgender participants. One study did not report participant demographics, such as gender or age (Jones et al., 2000).

**Figure 2:**
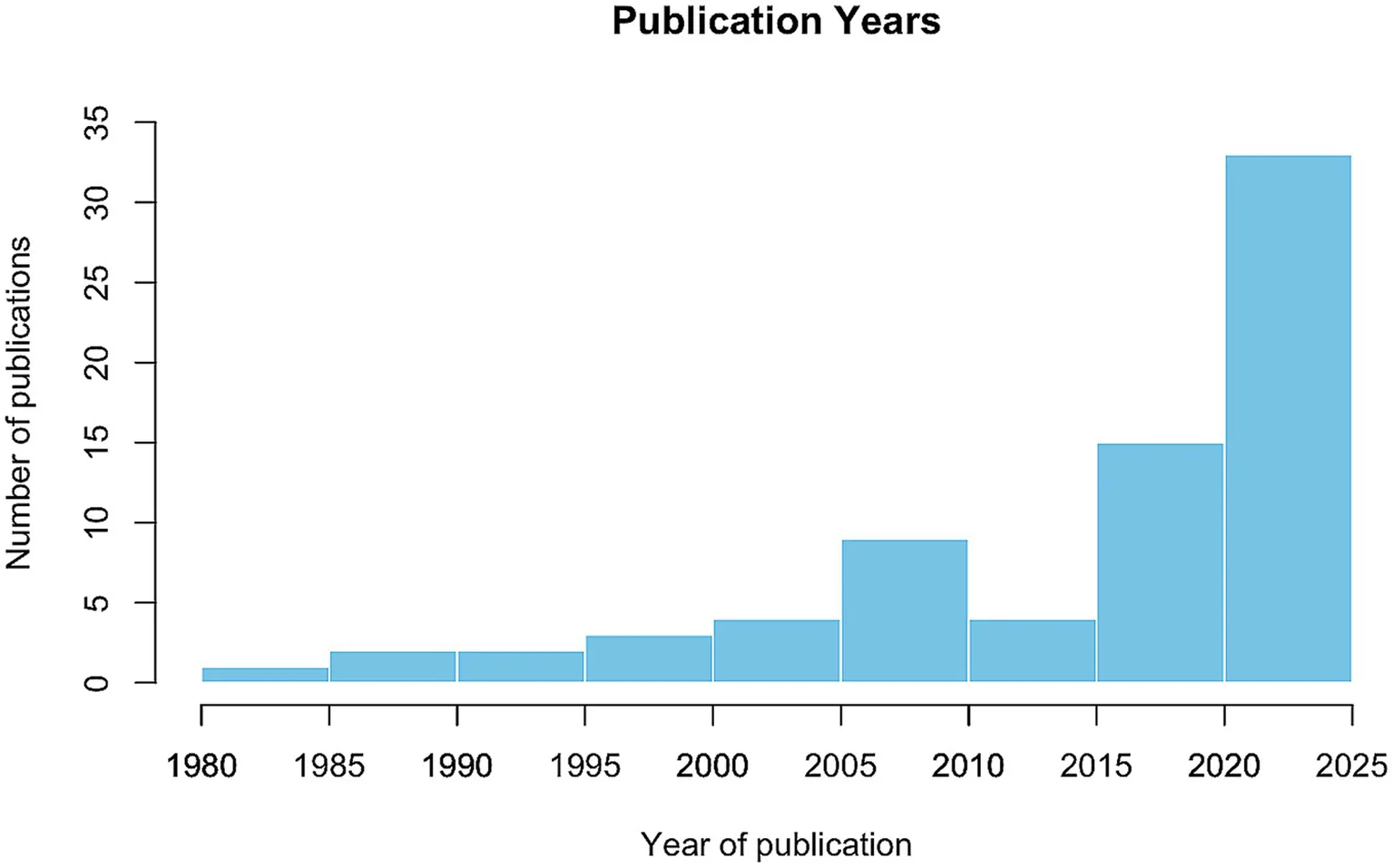
Number of publications involving ESMs per calendar year.

The studies were published between 1984 and 2025, with most being published in the last 15 years (Figure 2). Although there have been relatively few publications, the use of ESM in sport psychology has been recorded globally. From the final collection of 73 studies, 37 featured participants from Europe, 29 featured participants from North America, four enrolled participants from Asia, three involved participants from Oceania, and one had participants from Africa (sums to 74, as one study recruited from both the Netherlands and Australia). The sample sizes ranged from one to 401 participants, with a mean of 72 participants.

The included studies encompassed a wide range of sports and varying levels of competition. Half of the studies featured a single sport (*n* = 37), whereas most of the remaining studies featured two or more sports (*n* = 33). Three studies did not report the sport type as recruitment consisted of athletes from a sport medicine or sport psychology clinic (Trbovich et al., 2021; Ekelund et al., 2025), and student spectators during an unspecified national championship season (Riley et al., 2021). Across all studies, the most popular mentioned sport was soccer (i.e., football; *n* = 19), followed by swimming and/or diving (*n* = 13), basketball (*n* = 12), track and field and/or cross country (*n* = 11), and indoor or beach volleyball (*n* = 9). The sport performance context varied greatly. The most featured level of sport was at the national and/or international level (*n* = 27), where adult (*n* = 19) and youth athletes (*n* = 8) would compete at national and/or international competitions or within national and/or international leagues. Closely following was participation at the recreational level (*n* = 19), where adults (*n* = 11) and youth (*n* = 8) engaged in sport activities in a leisurely setting. Many studies featured sports at the professional level (*n* = 13), both in adult (*n* = 11) and youth (i.e., academy; *n* = 2) leagues. Eleven studies used ESMs in a collegiate or university setting, ten studies featured regional-to-national settings, and two included a mix of regional, national, and/or international levels. Two studies did not disclose the level of sport organization as athletes were recruited from sport medicine clinics (González-Barato et al., 2021; Trbovich et al., 2021).

### Study design features

The broad criteria used in our search were reflected in the included studies through a range of methodological choices. Only a third of the studies employed three or more assessments in a day (*n* = 26). Instead, many studies sampled experiences twice daily (*n* = 14), often organized as morning and evening questionnaires or before and after an event, whereas most used a “daily diary” structure (*n* = 34), with questionnaires employed once every 1–3 days (sums to 74, as one study sampled athletes daily and coaches twice daily). A fixed sampling scheme was the most common (*n* = 30), closely followed by the event-contingent sampling scheme (*n* = 22). The remaining studies used a fully random scheme (*n* = 8) or a semi-random scheme (*n* = 10). Three studies did not report the type of sampling scheme (Czajkowska et al., 2019; Sun et al., 2021; Wilson et al., 2025a).

We also coded the context in which the ESMs were conducted; specifically, whether the signals were sent before, during, or after the targeted sporting event or activity. Ten studies explicitly sampled experiences during the event of interest: six examined spectators’ experiences as they watched sporting events, while another four engaged with participants during water breaks in soccer games (Imtiaz et al., 2016), at defined points while running a marathon (Schüler and Brunner, 2009), between rapids while whitewater kayaking (Jones et al., 2000), or while climbing in the Himalayas (Fave et al., 2003). The other 61 studies used their various schemes to sample experiences in the hours before and/or after the events in question. Three studies did not specify when the ESM signals were scheduled (i.e., Czajkowska et al., 2019; Sun et al., 2021; Wilson et al., 2025a).

The included studies used a variety of instruments to collect data. The most popular method involved distributing physical booklets containing multiple pages of questionnaires (*n* = 28), and researchers employed various strategies to encourage completion. Most prominently, researchers distributed pagers or wristwatches that beeped to indicate when to complete a questionnaire (*n* = 10), had researchers supervise booklet completion (*n* = 5), collected completed booklets periodically during the study (e.g., once per week; *n* = 3), or called or texted participants with a reminder (*n* = 3). The second most popular method involved digital applications (*n* = 24) that sent push notifications prompting participants to complete measures within the application. These apps were mainly downloaded to participants’ smartphones, although specific studies distributed digital tablets (Ye et al., 2022) or personal digital assistants (Rumbold et al., 2020). Similarly, 16 studies asked participants to complete measures via a web-based survey platform. Six studies used text messages to send individual survey links to participants at each collection point. In contrast, others used a recurring link with reminders sent via email or text (some did not specify the reminder method). Finally, three studies collected data by reading each questionnaire item aloud to the participant and recording their responses. This verbal method was used when other methods were not feasible, such as when participants were running a marathon (Schüler and Brunner, 2009). One study used a combination of survey links sent via text message and audio diary recordings (Kent et al., 2025). One study did not report the data collection method (Czajkowska et al., 2019).

Considering the practical demands of collecting repeated measures of often momentary experiences, many studies developed measures tailored to their aims. Specifically, 16 studies exclusively used purpose-created sampling questionnaires, another 31 used a mix of novel and previously validated measures, while 26 used only previously validated measures. These classifications exclusively applied to the questionnaires used for the repeated measurements of the study and not to the baseline assessments, if conducted. Twenty studies used previously validated scales or subscales, 14 used measures with no identified psychometric support, and two used items rationalized by literature (without presenting psychometric evidence). The remaining 37 studies used mixed measurement approaches. Across all 73 included studies, most used at least one previously validated measure (*n* = 56), but nearly half included at least one measure for which no psychometric support was identified (*n* = 32). Relatively few studies reported study-specific evidence of reliability and/or validity for the repeated measures used in the study (*n* = 11), and 15 studies justified item selection through prior literature without presenting psychometric evidence.

### Areas of research

The studies were inductively grouped into six distinct areas of research based on their primary focus: stress and emotion, group dynamics, motivation, participants’ experiences across different activities, spectators’ experiences, and medical symptomatology. Approximately half the studies (*n* = 37) examined athletes’ (*n* = 33) or coaches’ (*n* = 4) experiences related to stress and emotion. This group was large enough to split into four subgroups. The largest subgroup (*n* = 19) investigated constructs of stress and/or emotion explicitly, almost always grounded in theories of cognitive appraisal and coping (Lazarus and Folkman, 1984; Lazarus, 1999), including the identification of primary or secondary appraisals and coping processes, and the consequent responses. Whereas all studies in this group leveraged concepts of appraisal and coping, some focused more on stress responses while others focused on emotional experiences. For example, Cerin and Barnett (2011) asked martial artists to complete measures five times per day in the week leading up to a competition to understand the relationships between their momentary concerns, appraisals of those concerns, and consequent affective states. Alternatively, Levy et al. (2009) asked an elite coach to use 28 consecutive daily diaries to report on perceived stressors, coping responses, and ratings of coping effectiveness. The second subgroup of studies on stress and emotion (*n* = 5) examined constructs that could modify processes of appraisal and coping, including self-compassion (Doorley et al., 2022; Röthlin et al., 2023), mindfulness (Zhang et al., 2024), savouring (Doorley and Kashdan, 2021) or emotional regulation (Lulu et al., 2025).

The final two subgroups of studies related to stress and emotion that were not explicitly tied to appraisal and coping represented athletic recovery and topics adjacent to stress and coping. Studies on athletic recovery (*n* = 7) took an applied perspective to examine associations between stress (i.e., athletic training), recovery, and psychological mediators of those relationships. Prominent mediators of the training-recovery relationship included sleep (Andolsek and Čater, 2025), recovery self-regulation (Wilson et al., 2025b), and experiences of psychological detachment (i.e., switching off; Balk et al., 2017; Balk et al., 2019; Balk et al., 2021; Balk and de Jonge, 2021; Descôtes et al., 2025). The final subgroup of stress and emotion-adjacent topics (*n* = 6) included experiences of loneliness (Luo et al., 2023), general mental health (Sun et al., 2021), and four studies examining athletes’ experiences of flow state while rock climbing (Fave et al., 2003), marathon running (Schüler and Brunner, 2009), whitewater kayaking (Jones et al., 2000), and participating in basketball and golf (Stein et al., 1995).

Beyond the predominant group of stress and emotion-focused studies, two other main psychological constructs were examined on within or between-day scales. In the first group, six studies examined daily or weekly variation of group dynamics in sport, including specific topics of youth athletes’ social identity (Herbison et al., 2021), role perceptions (Wilson et al., 2025a), prosocial and antisocial behaviours towards teammates (Benson and Bruner, 2018), interpersonal emotional regulation (Tamminen et al., 2019; Rumbold et al., 2025) or social comparisons between teammates (Diel et al., 2021). Notably, five of these six studies used emotion-related variables as the primary outcomes of their social constructs. In the second group, four studies examined elements of motivation, including achievement goals (Conroy et al., 2008), motivational predictors of collegiate athletes’ physical activity (Reifsteck et al., 2021), and psychological needs satisfaction among youth gymnasts (Gagné et al., 2003) and rehabilitating athletes (Röthlin et al., 2023). Outside of these studies, several others grouped elsewhere drew on concepts of autonomy and supportive coaching (Balk et al., 2019) and psychological needs satisfaction (Kim and James, 2019, 2024) as secondary areas of interest.

The fourth group of studies (*n* = 8) examined the experiences of sport participants between activities. These studies generally compared qualities of engagement—including effort, enjoyment, interest, and concentration—between activities to define what separates sport from other pursuits. Most commonly, sport was compared against incidental physical activity (Jeckel and Sudeck, 2017; Koch et al., 2020) or other leisure pursuits (e.g., art, hobbies, games; Kleiber et al., 1986; Richards and Duckett, 1994; Shernoff and Vandell, 2007). Other comparisons included experiences of formal and informal sport (Chalip et al., 1984; Kirshnit et al., 1989) or adult-led versus peer-led youth sport (Imtiaz et al., 2016).

The fifth group of studies (*n* = 7) used ESMs to assess psychological experiences predicting or resulting from medical conditions. Specifically, these studies examined psychological dimensions of concussion recovery (Sufrinko et al., 2019; Trbovich et al., 2021; Ye et al., 2022), orthopedic injury (González-Barato et al., 2021), and menstrual cycle disorders (Czajkowska et al., 2019). Studies in this group integrated subjective psychological measures with objective ones such as sleep data (Trbovich et al., 2021) or neurocognitive testing (Sufrinko et al., 2019). One notable study in this area examined the feasibility of using ESMs to deliver a cognitive behavioural therapy intervention to treat anxiety and depression (Ekelund et al., 2025), while another predicted injury risk using prior self-reported psychological data (e.g., recovery, motivation, mood; Neumann et al., 2024).

The sixth and final group of studies (*n* = 11) pertained to spectators’ experiences of engaging with sports. Within this group, one subset of studies (*n* = 5) explored how behaviours of consuming alcohol or gambling on sports were influenced by the psychological experiences—such as emotional dysregulation, rumination, and anger (Riley et al., 2021; Freisthler et al., 2023)—or social contexts (Anderson-Luxford et al., 2023; Pennay et al., 2023) associated with spectating sports. The other six studies explored characteristics of various cognitive and/or emotional experiences of watching sports, including nostalgia (Hungenberg et al., 2020), entertainment (David et al., 2008; Götz et al., 2020), and vicarious achievement or needs fulfillment (Dwyer et al., 2016; Kim and James, 2019, 2024).

## Discussion

The findings of this scoping review summarize the use of ESMs within empirical research in sport psychology. Our review included 73 studies, which is much smaller than similar reviews in comparable areas, such as health psychology (e.g., 633 studies; Perski et al., 2022). We described the contexts (e.g., populations, sports), designs (e.g., sampling frequency and scheme), and topical themes featured in this body of research. We found that ESMs have most often been used to represent Western (e.g., North America and Europe) sport participants’ perspectives across a range of sporting levels. ESMs have been employed across a wide variety of designs and topics, with an emphasis on constructs of stress and emotion. To connect these findings to their broader implications, we discuss the relative strengths and weaknesses of this body of research across three main features of ESM use in sport psychology: the contexts in which ESMs have been applied, the study designs used to capture momentary experiences, and the research topics that have been examined or remain underdeveloped.

### Context

Although the included studies covered a variety of contexts overall, there was a tendency to focus on certain areas. For instance, our findings align with broader patterns of overrepresentation of participants from Europe and North America, as well as of sports such as soccer, basketball, and volleyball (Baker et al., 2020). This concentration is important because ESMs are intended to capture contextual variation in momentary experiences, which may be shaped by cultural systems, sport structures, and norms surrounding, for example, coaching approaches, competitive structures, or parental involvement. Similarly, the over-representation of team-based sports with intermittent competitive play (e.g., soccer, basketball, volleyball) may offer different opportunities or experiences compared to more continuous or individual sports. Although the existing literature does not yet allow for direct comparisons across these contexts, future research should examine how sport type and sociocultural context influence momentary experiences in sport.

Notably, most studies focused on athletes (or otherwise direct participants in sport). Previous studies have noted that the use of ESMs in sport research may be limited by interference from completing self-report measures during game or practice participation (Reifsteck et al., 2021). Many studies navigated this potential interference by using pre and post-game assessments (Cerin and Barnett, 2006) or examining experiences ‘outside’ of the arena (e.g., interpersonal emotion regulation among teammates; Tamminen et al., 2019). However, we also found several creative solutions to ascertain participants’ “in-game” experiences, including sampling youth athletes during water breaks (Imtiaz et al., 2016), sampling whitewater kayakers at natural resting points between rapids (Jones et al., 2000), or running alongside athletes in a marathon race (Schüler and Brunner, 2009). Thus, while the use of ESMs during sport activities presents certain challenges, these complexities should not preclude future investigations of real-time participation experiences.

In contrast to studies that focus on athletes’ perspectives, relatively few studies have examined the experiences of coaches, parents, or spectators. Only five studies examined coaching experience to any degree; all were published recently and focused on elements of the stress appraisal and coping process. One study examined parents’ behaviours while spectating the Super Bowl (Freisthler et al., 2023), and another sampled their perceptions of stressors related to their child’s training (Hayward et al., 2017). Notably, an absence of investigations of supporters is not unique to sport—health research has also seen a relative lack of ESM studies examining caregiver experiences (c.f., Singh et al., 2025). However, the lack of sport research in this area was surprising not only in light of the importance of coaches’ and parents’ experiences within sport (Côté et al., 2020) but also considering that they should be more free than athletes to engage with ESMs during sporting events. The latter point is reinforced by the subgroup of studies that examined fans’ experiences of watching sporting events. Although some of these studies examined “in-game” predictors of spectators’ health perceptions and behaviours, several examined emotional experiences (Götz et al., 2020) that could be insightful in understanding how coaches and parents experience sporting events, and whether those experiences influence their future perceptions, behaviours, or interactions with athletes. Coaches, spectators, and parents undeniably shape the athlete experience in sports, so further research should explore how ESMs may be used to examine how the emotions and behaviours of individuals in these roles influence daily experiences of sport.

### Study design

Our findings represented a wide range of designs under the umbrella of ESMs. Only 16 studies used a “traditional” ESM design featuring multiple signals per day sent on a random or semi-random schedule (Myin-Germeys and Kuppens, 2022). There was a total of 18 studies that implemented a random or semi-random schedule; however, two of these studies did not send multiple signals a day (Sitch and Day, 2015; Kim and James, 2024). Instead, most involved sampling experiences approximately once (i.e., “daily diary” structure) or twice per day (e.g., morning and evening; before and after practice). These different designs favour the measurement of constructs at different temporal scales, rather than being inherently better or worse. The use of before/after practice or competition designs favours overall perceptions of an event, as opposed to “in the moment” experiences. Similarly, daily or morning/evening designs suit constructs that are experienced or evolve on a day-to-day basis, such as mood or organizational stress, as opposed to the more momentary emotions or reactions that evolve throughout a day and would be better captured through higher-frequency, random sampling. While these designs may all be considered variants of ESMs, their relative use suggests that psychological research in sport has only superficially examined the momentary contexts and processes that constitute sport experiences.

Our findings also indicate a similar preference for measures undertaken around a sporting event rather than during it. Our review identified 10 studies that sampled experiences *during* sport, of which only four considered participants’ perspectives. On the surface, health behaviour research shows a similar pattern, with studies struggling to assess specific events. Specifically, Perski et al. (2022) found that only 5% of 633 health psychology studies used event-contingent (rather than time-based) signalling. However, whereas the main challenge in health research is “finding” instances of smoking or substance use, the challenge in sport comes from integrating measurement within the experience in question. On one hand, the findings of our review suggest that psychological sport research may still be reliant on retrospective or summary perspectives of experiences to a degree, even if only considering the duration of a game or practice. On the other hand, these findings suggest that 63 studies found meaning in sampling perspectives outside of the “arena,” which contradicts the suggestion that ESMs are not suitable for sport research because they are difficult to implement during sport activity (Reifsteck et al., 2021). The findings of this review provide examples of studies that have successfully used ESMs during practice/competitions, as well as examples of how ESMs may be meaningfully implemented outside those settings.

Our findings indicate that the specific data collection tools and measures used within ESMs have continued to evolve over time, according to the research context. We found that a variety of tools (e.g., physical booklets, smartphone apps) and measures (both established and novel) were used in ESM-based sport psychology research. Whereas this variation likely follows characteristics of the specific design, population, and study resources, sport psychology researchers should consult existing guidelines regarding the design of ESM-based studies (e.g., Kuppens et al., 2022; Myin-Germeys and Kuppens, 2022). Similarly, a large proportion of the studies used adapted, novel, or mixed measurement approaches, likely to reduce participant burden when completing repeated measures (Myin-Germeys and Kuppens, 2022). Although single-item measures can be valid predictors compared to multidimensional alternatives (Song et al., 2023), our findings suggest that support for these measures was reported inconsistently across the included studies. Most studies included at least one previously validated measure, but relatively few reported study-specific evidence of reliability and/or validity for the repeated measures used. Several studies included measures for which no psychometric support was identified. These measures often appeared to capture specific contextual, situational, or discrete information, but their use nevertheless highlights the need for researchers to more explicitly justify their measurement choices in future ESM-based sport psychology research.

### Areas of research

When grouped according to thematic areas of research, most included studies used ESMs to examine concepts related to stress and emotion. These findings are not surprising, as stress and emotions are prominent topics in sport psychology (e.g., Simpson et al., 2024) and they involve momentary experiences that are well-suited for exploration through ESMs, as seen by their common investigation in other areas of psychology (e.g., Boemo et al., 2022; Perski et al., 2022). Even among studies that defined sport experiences in relation to other activities, measures of affect (e.g., mood, enjoyment) were common. As a result, our findings represent a useful body of research describing daily or momentary sport-related experiences of positive and negative affect, as well as stress, its antecedents, and its consequences.

Beyond these strengths, we identified several areas of research that were surprisingly underdeveloped. For instance, motivation, self-regulation, enjoyment, and group dynamics are primary topics of investigation within sport psychology (Schüler et al., 2023) that have been frequently examined using ESMs in other areas of psychology (e.g., Votaw and Witkiewitz, 2021; Perski et al., 2022; Langener et al., 2023). However, we found only four studies that focused on motivational processes and six that examined interpersonal dynamics. While processes of self-regulation were implicitly examined in several studies of emotions or anxiety, and in one study of the self-regulation of recovery (Wilson et al., 2025b), the behavioural consequences of self-regulation were examined only through sport spectators’ health-related behaviours (e.g., gambling, alcohol consumption). Motivation, self-regulation, and group dynamics each involve momentary experiences, decisions, and interactions that may be misrepresented if aggregated through traditional global or retrospective methods. Therefore, greater use of ESMs in these areas could enhance understanding of how these constructs fluctuate across specific sport contexts, and how they may be supported within athletes’ daily sport environments.

It is important to note that our findings may be limited by methodological choices around search terms and inclusion criteria. For instance, we found few studies that examined athletes’ perceptions alongside physical training variables (c.f., Neumann et al., 2024). While such studies would meet our inclusion criteria (e.g., Kölling et al., 2015), they either did not refer explicitly to ESMs (or any related method) or the domain of psychology. Considering that we aimed to examine the specific use of ESMs, it was beyond our scope to search training-based studies for daily, self-report variables. However, this parallel body of research indicates that greater interdisciplinary collaboration is needed, especially as expanded use of wearables to study athlete training offers substantial potential for intensive longitudinal psychological studies. We deliberately excluded studies that focused exclusively on accelerometer data or biological measures, as they did not directly assess psychosocial outcomes, which are central to our study focus. Similarly, we excluded studies that relied on narrative or reflective open-ended journal entries, as these methodologies stray away from the repeated, real-time measurement of ESMs.

## Conclusion

ESMs are well-suited to capture the dynamic changes in experience and context that occur from moment to moment in sport. Our findings indicate that sport psychology research has thus far made limited use of ESMs. Where they have been implemented, ESMs have largely focused on participants’ experiences of stress and emotions, using (near-)daily methods. Although these tendencies represent strengths in the extant literature, they also indicate the potential for future research to explore the experiences of non-participating actors (e.g., coaches, parents) and to examine processes that unfold over finer temporal scales than from beginning to end of the day. By capturing real-time behaviours, emotions, and attitudes, as well as environmental factors and external stressors, researchers can gain a more comprehensive understanding of the daily demands on athletes, coaches, parents, and spectators.

## Data Availability

The datasets presented in this study can be found in online repositories. The names of the repository/repositories and accession number(s) can be found in the article/Supplementary material.

## Footnotes

1
https://osf.io/knxgw/?view_only=6b23569df1cd49ae8288182746099152
