# Supplementary material for: Examining the use of experience sampling methods in sport psychology research: a scoping review
Source: Front Psychol. 2026 Aug 10;17:1860704. doi: 10.3389/fpsyg.2026.1860704 (PMC13500244; doi:10.3389/fpsyg.2026.1860704)
Supplement: Supplementary file 1 [file Data_Sheet_1.docx]

| Author | Population | Sport | Sampling Scheme | Sampling Frequency | Research Topic |
| --- | --- | --- | --- | --- | --- |
| Anderson-Luxford et al. (2023) | Spectator | Australian football | Event-contingent | 2/week | Spectator experiences (alcohol & social context) |
| Andolšek & Čater (2025) | Athlete | Handball | Fixed | 1/day | Stress & emotions (recovery) |
| Balk & de Jonge (2021) | Athlete | Soccer, swimming, Australian football, handball, water polo, beach volleyball, speed skating, archery | Fixed | 2/day | Stress & emotions (recovery) |
| Balk et al. (2017) | Athlete | Water polo, volleyball, beach volleyball, soccer, basketball, archery, para-cycling, and para-athletics | Fixed | 2/day | Stress & emotions (recovery) |
| Balk et al. (2019) | Coach and athlete | Soccer, swimming, Australian football, handball, water polo, beach volleyball, speed skating, archery | Fixed | Coaches 2/day, athletes 1/day | Stress & emotions (recovery) |
| Balk et al. (2021) | Athlete | Swimming, volleyball, and water polo | Fixed | 2/day | Stress & emotions (recovery) |
| Benson & Bruner (2018) | Athlete | Hockey | Fixed | 1/day | Social dynamics  (organizational citizenship behaviours) |
| Cerin et al. (2001) | Athlete | Tae kwon do | Semi-random | 3/day before event, 1/ day of competition, 1/day after competition | Stress & emotions (emotions) |
| Cerin (2004) | Athlete | Tae kwon do | Semi-random | 3/day before competition, 1/day of competition | Stress & emotions (anxiety) |
| Cerin & Barnett (2006) | Athlete | Tae kwon do and karate | Random | 5/day | Stress & emotions (emotions) |
| Cerin & Barnett (2011) | Athlete | Tae kwon do and karate | Random | 5/day | Stress & emotions (emotions) |
| Chalip et al. (1984) | Athlete (high school students participating in sport activity) | Tennis, baseball, basketball, softball, gymnastics, swimming, handball, track, weightlifting, golf, badminton, skiing | Random | ~9/day | Sport comparison (sport vs. physical activity) |
| Conroy et al. (2008) | Athlete | Track and field | Fixed | 1/day | Motivation (achievement goal theory) |
| Czajkowska et al. (2019) | Athlete | Gymnastics | Unreported | Unreported | Medical symptoms (premenstrual symptoms) |
| Dagrou & Szabo (1998) | Athlete | Track and field | Fixed | 1/day | Stress & emotions (anxiety) |
| David et al. (2008) | Spectator | American football | Event-contingent | 30/day | Spectator experiences (entertainment) |
| Descôtes et al. (2025) | Athlete | Ice hockey | Fixed | 1/day | Stress & emotions (recovery) |
| Didymus & Fletcher (2012) | Athlete | Swimming | Fixed | 1/day | Stress & emotions (organizational stressors) |
| Diel et al. (2021) | Athlete | Swimming, track and field | Event-contingent | 1/week | Social dynamics (social comparisons) |
| Doorley & Kashdan (2021) | Athlete | Soccer, volleyball, swimming and diving, basketball, cross country and track and field, lacrosse, and softball | Fixed | 1/day | Stress & emotions (savouring) |
| Doorley et al. (2022) | Athlete | Soccer, cross country and track and field, swimming, basketball, softball, and field hockey | Fixed | 1/day | Stress & emotions (self-compassion) |
| Dwyer et al. (2016) | Spectator | NFL and fantasy football | Event-contingent | 3/week | Spectator experiences (BIRG, CORF) |
| Ekelund et al. (2025) | Athlete | Mixed sports, not specified | Semi-random | 6/day | Medical symptoms (mental illness) |
| Fave et al. (2003) | Athlete | Rock climbing | Semi-random | 4-6/day | Stress & emotions (flow state) |
| Freisthler et al. (2023) | Parent | American football | Fixed | 3/day | Spectator experiences (parent aggression) |
| Gagne et al. (2003) | Athlete | Gymnastics | Event-contingent | 2/practice | Motivation (self-determination theory) |
| Gaudreau et al. (2010) | Athlete | Golf | Event-contingent | 1/round | Stress & emotions (appraisal & coping) |
| González-Barato et al. (2021) | Athlete | Soccer, basketball, frisbee, judo, and handball | Event-contingent | 1/day | Medical symptoms (injury monitoring) |
| Götz et al. (2020) | Spectator | Soccer | Event-contingent | 3/day | Spectator experiences (emotions) |
| Hayward et al. (2017) | Parent and athlete | Swimming | Event-contingent | 1/day | Stress & emotions (stressors) |
| Herbison et al. (2021) | Athlete | Ice hockey | Fixed | 1/day | Social dynamics (social identity) |
| Hungenberg et al. (2020) | Spectator | Baseball | Fixed | 7/game | Spectator experiences (nostalgia) |
| Hyoun et al. (2023) | Spectator | Hockey and basketball | Fixed | 2/day | Spectator experiences (gambling & dysregulation) |
| Imtaiz et al. (2016) | Athlete | Soccer | Event-contingent | 2/training session | Sport comparison (adult-led vs. youth-led sport) |
| Jeckel & Sudeck (2017) | Athlete | Sport activities, not specified | Fixed | 4/day | Sport comparison (sport vs. physical activity) |
| Jones et al. (2000) | Athlete | Kayaking | Event-contingent | 8/event | Stress & emotions (flow state) |
| Kent et al. (2025) | Athlete | Badminton | Semi-random | 3/day | Stress & emotions (appraisal & coping) |
| Kim & James (2019) | Athlete and spectator (university students participating & spectating) | Mixed sports, not specified | Event-contingent | Questionnaire A 2/3 weekly, Questionnaire B 1/day | Spectator experiences (self-determination theory) |
| Kim & James (2024) | Athlete and spectator (university students participating & spectating) | Mixed sports, not specified | Semi-random | 2-3/week | Spectator experiences (self-determination theory) |
| Kirshnit et al. (1989) | Athlete (elementary students participating in sport activity) | Organized sports (Little League and interscholastic athletics) | Random | 7/day | Sport comparison (formal vs. informal sport) |
| Kleiber et al. (1986) | Athlete (high school students participating in sport activity) | Mixed sports, not specified | Fixed | 8/day | Sport comparison (adult-led vs. youth-led sport) |
| Koch et al. (2020) | Athlete (participating in sport activity) | Mixed sports, not specified | Event-contingent | 4-17/day | Sport comparison (adult-led vs. youth-led sport) |
| Kratz et al. (1997) | Athlete | Rugby, sailing | Event-contingent | 1/sport activity | Stress & emotions (participation experience) |
| Levy et al. (2009) | Coach | Aquatic sport, not specified | Fixed | 1/day | Stress & emotions (stressors) |
| Lulu et al. (2025) | Athlete | Shooting | Fixed | 2/day | Stress & coping (emotional regulation) |
| Luo et al. (2023) | Athlete | Honor of Kings and League of Legends | Fixed | 1/day | Stress & emotions (loneliness) |
| Neumann et al. (2024) | Athlete | Soccer | Fixed | 2/day | Medical symptoms (injury prediction) |
| Nicholls & Polman (2007) | Athlete | Rugby | Fixed | 1/day | Stress & emotions (stressors) |
| Pennay et al. (2022) | Spectators | Australian football | Event-contingent | maximum 10/day | Spectator experiences (alcohol & social context) |
| Polman et al. (2007) | Athlete | Rugby | Fixed | 1/day | Stress & emotions (mood) |
| Potts et al. (2024) | Coach | Track and field, race walking, gymnastics, soccer, field hockey | Event-contingent | 1/day | Stress & emotions (wellbeing) |
| Reeves et al. (2011) | Athlete | Soccer | Fixed | 1/day | Stress & emotions (stressors) |
| Reifsteck et al. (2021) | Athlete | Baseball, softball, basketball, tennis | Random | 4/day | Motivation (physical activity intentions) |
| Richards & Duckett (1994) | Athlete (elementary students participating in sport activity) | Sports, not specified | Random | 7/day | Sport comparison (sport vs. physical activity) |
| Riley et al. (2021) | Spectator | Unreported | Fixed | 1/day | Spectator experiences (alcohol and stress) |
| Röthlin et al. (2023) | Athlete | 26 different sports; three most common sports were soccer, athletics (track and field) and floorball | Event-contingent | 2/day | Stress & emotions (self-compassion) |
| Röthlin et al. (2025) | Athlete | 29 different sports; most common were soccer, volleyball, handball | Semi-random | 3/day | Motivation (self-determination theory) |
| Rumbold et al. (2020) | Athlete | Rugby | Semi-random | 2/day | Stress & emotions (organizational stressors) |
| Rumbold et al. (2022) | Athlete | Soccer | Event-contingent | 1/week | Stress & emotions (emotional contagion) |
| Rumbold et al. (2023) | Coach | Australian football, badminton, basketball, diving, field hockey, golf, gymnastics, martial arts, netball, rugby, soccer, swimming, tennis, track and field, triathlon, volleyball, and water polo | Semi-random | 2/day | Stress & emotions (emotional exhaustion) |
| Rumbold et al. (2025) | Athlete | Soccer | Event-contingent | 2/day | Social dynamics (interpersonal regulation) |
| Schuler & Brunner (2009) | Athlete | Marathon running | Event-contingent | 4/race | Stress & emotions (flow state) |
| Shernoff et al. (2007) | Athlete | Combined organized sports and informal/solitary sport | Random | 5/day | Sport comparison (youth sport vs. other activities) |
| Sitch & Day (2015) | Athlete | Judo | Random | 1/day | Stress & emotions (appraisal & coping) |
| Stein et al. (1995) | Athlete | Tennis, basketball, golf | Event-contingent | 2/day | Stress & emotions (flow state) |
| Sufrinko et al. (2019) | Athlete | American football,  soccer, ice hockey, basketball, lacrosse, competitive cheer/dance | Fixed | 3/day | Medical symptoms (concussions) |
| Sun et al. (2021) | Athlete | Soccer | Unreported | 1/day | Stress & emotions (mental health) |
| Tamminen et al. (2019) | Athlete | Ice hockey, field hockey, volleyball, basketball | Fixed | 1/day | Social dynamics (interpersonal regulation) |
| Trbovich et al. (2021) | Athlete | Unreported | Fixed | 3/day | Medical symptoms (concussions) |
| Wilson et al. (2025a) | Athlete | Ice hockey | Unreported | 1/day | Social dynamics (role perceptions) |
| Wilson et al. (2025b) | Athlete | Road cycling, track cycling, mountain biking, triathlon | Semi-random | 8/day | Stress & emotions (recovery) |
| Ye et al. (2022) | Athlete | Soccer | Fixed | 1/day | Medical symptoms (concussions) |
| Zhang et al. (2024) | Athlete | Synchronized swimming, trampoline, weightlifting, table tennis, free combat, water polo, gymnastics, track and field, diving, martial arts, and swimming | Event-contingent | 1/day; 3x/week | Stress & emotions (mindfulness) |
