# Supplementary material for: Examining the use of experience sampling methods in sport psychology research: a scoping review
Source: Front Psychol. 2026 Aug 10;17:1860704. doi: 10.3389/fpsyg.2026.1860704 (PMC13500244; doi:10.3389/fpsyg.2026.1860704)
Supplement: Supplementary file 2 [file Supplementary_File_1.docx]

**Scoping Review Protocol**

**Title**: Examining the Use of Experience Sampling Methods in Sport Psychology Research: A Scoping Review.

**Authors**: Mara L. L. Majer^1^, Mitchell C. Profeit^1^, Luc J. Martin^1^, Jean Côté^1^, Andre Spendlove^1^ and Stuart G. Wilson^1^

^1^ School and Kinesiology & Health Studies, Queen’s University

**Corresponding Author**:

Mara L. L. Majer

School of Kinesiology & Health Studies, Queen’s University

28 Division Street, Kingston, Ontario, Canada

K7L 3N6

Email: [19mllm@queensu.ca](mailto:19mllm@queensu.ca)

**Introduction**

Experience sampling methods provide unique and powerful insight into the moment-to-moment experiences of daily life. For the purpose of this scoping review, experience sampling methods involve repeated questionnaires sent throughout a period of multiple days or weeks to measure quantitative or qualitative data about a participant’s immediate behaviors, feelings, and attitudes towards real-world events, environments, and/or relationships (Li et al., 2022; Rumbold et al., 2020; Trull & Ebner-Priemer, 2009). There is great variability in the day-to-day human experience, so sampling methods are used to answer research questions based on an individual’s emotions, thoughts, and behaviours surrounding daily inter-personal and intra-personal events through observational methods (Myin-Germeys et al., 2022). Although experience sampling methods are popular in psychological research more generally (Myin-Germeys et al., 2022), it is unclear how they have been implemented to study sport. The aim of this scoping review is to gather all existing literature using ESM in sport psychology and identify any gaps in the research to fuel future questions.

**Method**

This project will follow the guidelines for scoping reviews in sport and exercise psychology outlined by Sabiston et al. (2022), which build on more general frameworks outlined by Arksey and O’Malley (2005) and Levac et al. (2010). These guidelines used to guide this scoping review project include eight steps: (1) Create and consult with a stakeholder group, (2) Identify the research question, (3) Identify relevant studies, (4) Create and register a protocol, (5) Select and screen studies, (6) Chart the data, (7) Collate and summarize, (8) Re-consult stakeholders and identify implications (Sabiston et al., 2022). Within the following section, we briefly introduce each step.

**Stage 1: Create and Consult with a Stakeholder Group**

We created a stakeholder group representing researchers with expertise in a broad range of areas across sport psychology, including mental performance, expertise, positive youth development, coaching and leadership, and group dynamics. The lead researcher will meet with the stakeholder group before the project to discuss and specify the research questions, objectives, and considerations for exclusion criteria and characteristics to be coded. The stakeholder group will be comprised of the authorship team.

**Stage 2: Identify the Research Question**

Objective: To investigate how experience sampling methods (ESM) have been used in sport psychology research. Specific objectives of this scoping review will be to: (1) accumulate the existing studies using ESM in sport psychology, (2) understand the contexts in which ESM are being conducted, (3) identify what topics ESM has been used to measure, and (4) analyze the ESM study designs that have been used.

**Stage 3: Identify Relevant Studies**

Inclusion & Exclusion Criteria: We will include studies that represent original research published in peer-reviewed venues that use ESM or ecological momentary assessments in sport psychology. The inclusion criteria will consist of studies using questionnaires or diary entries to collect quantitative or qualitative data from athletes, coaches, fans, and/or parents before, during, and/or after competitive sport events. Exclusion criteria include any grey literature or non-peer-reviewed material, literature reviews, studies that use Descriptive Experience Sampling Methods (Dickens et al., 2018; Van Raalte et al., 2019), or data collection that is not related to a sport event, such as leisure activities, physical activity, or exercise.

Data Sources: The databases being used in this scoping review are SportDiscus, APA PyschInfo (via OVID), Sport Medicine & Education Index, and Web of Science. These search engines were discussed and selected in consultation with our departmental librarian, Michele Chittenden.

Search Strategy: The search string developed for this scoping review includes the following: "experience sampl*" OR "daily diar*" OR "ecological momentary assessment*" OR "ambulatory assessment*" OR "ecological momentary intervention*" OR "just in time adaptive intervention*" OR "JITAI*" AND "sport*"

**Stage 4: Select and Screen Studies**

Study selection will proceed in three stages. The study selection will begin with a broad search using the included terms in the specified databases. The search results will be uploaded to the Covidence search software (Covidence, 2023) and duplications will be removed. Two researchers will then independently review the title and abstract of the papers and decide whether to include or exclude them from the scoping review based on established criteria. Once the selection of studies has narrowed, the two researchers will review the full texts to select which articles follow our inclusion criteria. Any disagreements or discrepancies will be addressed with a third researcher from the team. We will use a PRISMA-ScR checklist and flow diagram to inform the process for reporting on the study selection (Tricco et al., 2018).

**Stage 5: Charting the Data**

The research team will develop an excel document to code specific terms in each paper. This chart will be developed in an ongoing and iterative way, and will include information such as the title, year, authors, sample demographics (e.g. age, sport, athlete, coach), study design (e.g. frequency of sampling), topic (e.g. emotions, stress), and context (e.g. pre-competition, post practice). Two researchers will conduct the search and then meet after reviewing ten papers to establish continuity between them, in line with the study's purpose.

**Stage 6: Collating and Summarizing the Results**

The recorded characteristics for each of the selected studies will be summarized numerically to characterize the scope and extent of studies within each category. Quantitative characteristics such as the number of papers included in the review, types of study designs, year of publication, and demographics of the study participants will be summarized as numerical counts. Qualitative data points such as topic of study (emotion, stress) and context (pre-competition, post practice) will be grouped and summarized numerically. After the data has been collecting and analyzed, the scoping review will discuss the implications these studies have on sport psychology research and identify gaps or questions for future research.

**Stage 7: Re-consultation with Stakeholders**

The research team will reconvene with stakeholders to review and discuss the findings from the scoping review. The stakeholders will help interpret the implications for gaps in the literature and identify future research questions.

**References**

Arksey, H., & O’Malley, L. (2005). Scoping studies: Towards a methodological framework. *International Journal of Social Research Methodology*, *8*(1), 19–32. <https://doi.org/10.1080/1364557032000119616>

Covidence systematic review software, Veritas Health Innovation, Melbourne, Australia. Available at [www.covidence.org](http://www.covidence.org)

Levac, D., Colquhoun, H., & O’Brien, K. K. (2010). Scoping studies: Advancing the methodology. *Implementation Science*, *5*(1), p#69. <https://doi.org/10.1186/1748-5908-5-69>

Li, Y.-M., Hachenberger, J., & Lemola, S. (2022). The Role of the Context of Physical Activity for Its Association with Affective Well-Being: An Experience Sampling Study in Young Adults. *International Journal of Environmental Research and Public Health*, *19*(17), 10468. <https://doi.org/10.3390/ijerph191710468>

Myin-Germeys, I., & Kuppens, P. (Eds.). (2022). *The open handbook of experience sampling methodology: A step-by-step guide to designing, conducting, and analyzing ESM studies* (2nd ed.). Center for Research on Experience Sampling and Ambulatory Methods Leuven.

Rumbold, J., Fletcher, D., & Daniels, K. (2020). An experience sampling study of organizational stress processes and future playing time in professional sport. *Journal of Sports Sciences*, *38*(5), 559–567. <https://doi.org/10.1080/02640414.2020.1717302>

Sabiston, C. M., Vani, M., de Jonge, M., & Nesbitt, A. (2022). Scoping reviews and rapid reviews. *International Review of Sport and Exercise Psychology*, *15*(1), 91–119. <https://doi.org/10.1080/1750984X.2021.1964095>

Tricco, A. C., Lillie, E., Zarin, W., O’Brien, K. K., Colquhoun, H., Levac, D., Moher, D., Peters, M. D. J., Horsley, T., Weeks, L., Hempel, S., Akl, E. A., Chang, C., McGowan, J., Stewart, L., Hartling, L., Aldcroft, A., Wilson, M. G., Garritty, C., … Straus, S. E. (2018). PRISMA Extension for Scoping Reviews (PRISMA-ScR): Checklist and Explanation. *Annals of Internal Medicine*, *169*(7), 467–473. <https://doi.org/10.7326/M18-0850>

Trull, T. J., & Ebner-Priemer, U. W. (2009). Using experience sampling methods/ecological momentary assessment (ESM/EMA) in clinical assessment and clinical research: Introduction to the special section. *Psychological Assessment*, *21*(4), 457–462. <https://doi.org/10.1037/a0017653>

Van Raalte, J. L., Vincent, A., & Dickens, Y. L. (2019). Dialogical Consciousness and Descriptive Experience Sampling: Implications for the Study of Intrapersonal Communication in Sport. *Frontiers in Psychology*, *10*, p#653. <https://doi.org/10.3389/fpsyg.2019.00653>
